# Supplementary figures and images for: Prognostic value of RNA methylation-related genes in gastric adenocarcinoma based on bioinformatics
Source: PeerJ. 2024 Feb 29;12:e16951. doi: 10.7717/peerj.16951 (PMC10909369; doi:10.7717/peerj.16951)

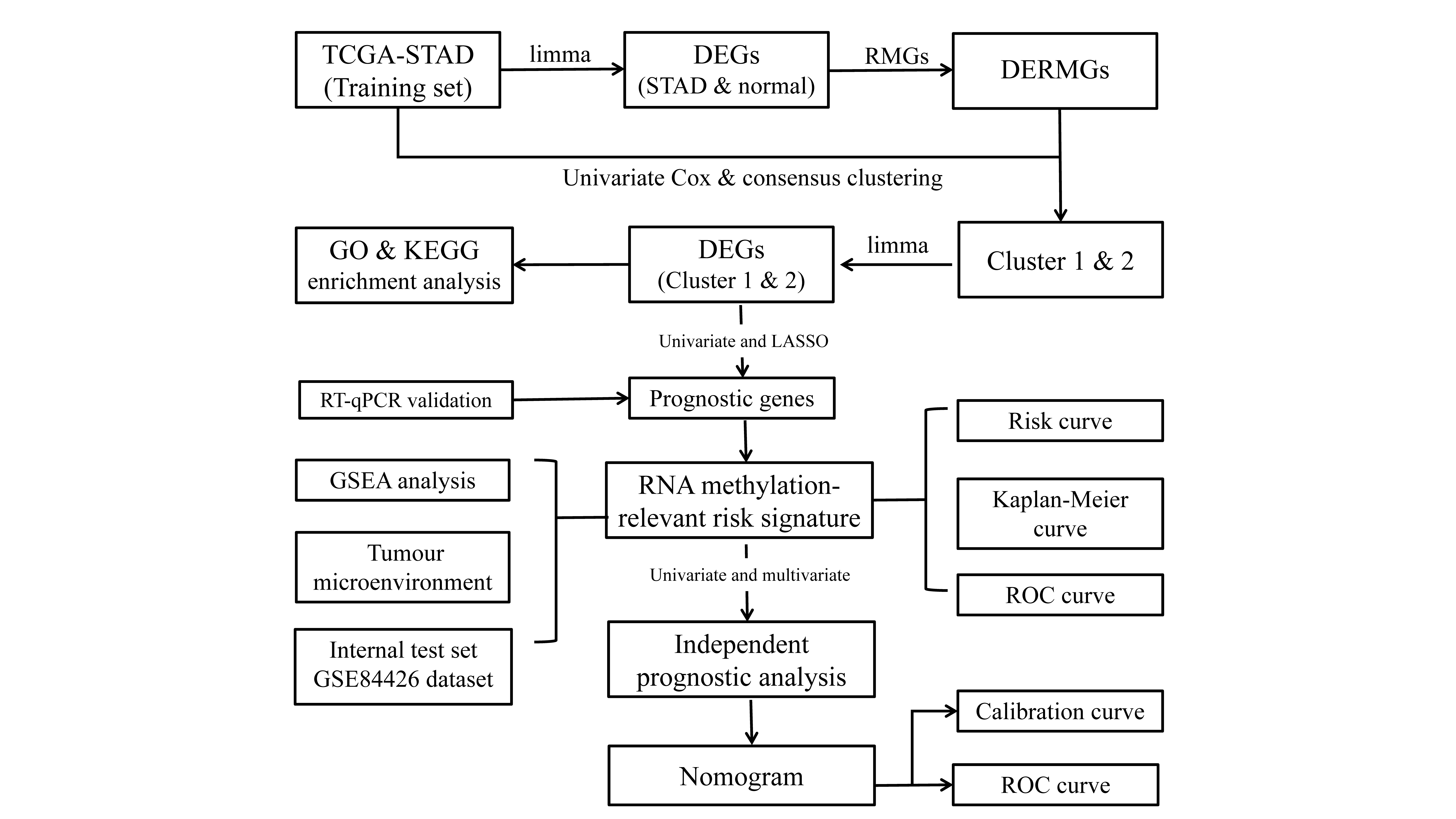

Supplement: Supplemental Information 1 [file peerj-12-16951-s001.png]

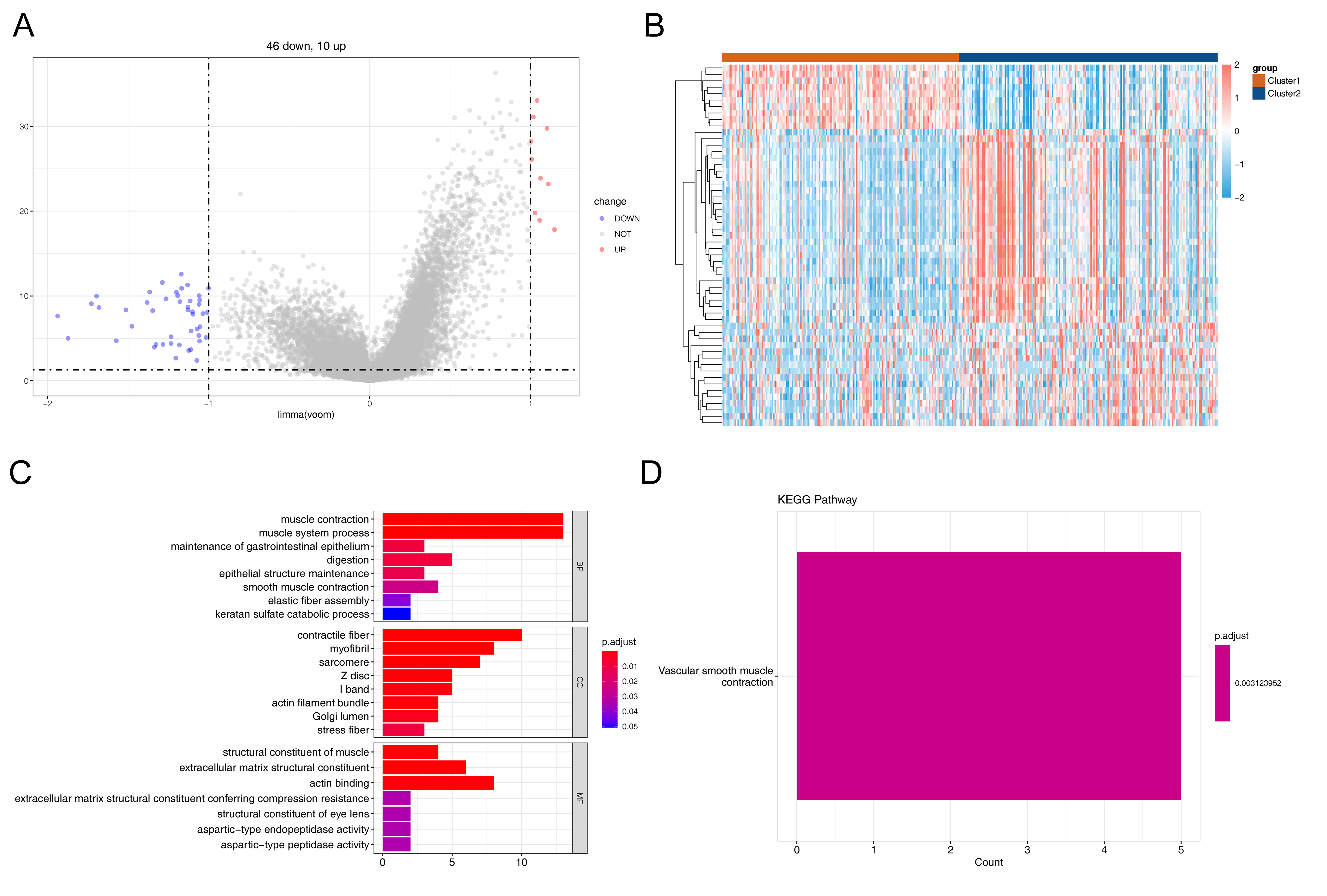

Supplement: Supplemental Information 2 — (A) The volcano map of DEGs between culster2 and culster1. (B) The heatmap of DEGs between culster2 and culster1. (C) The top 8 BP entries, top 8 CC entries, and 7 MF entries were enriched by DEGs between culster2 and culster1. (D) The KEGG pathways were enriched by DEGs between cluster2 and cluster1. [file peerj-12-16951-s002.png]

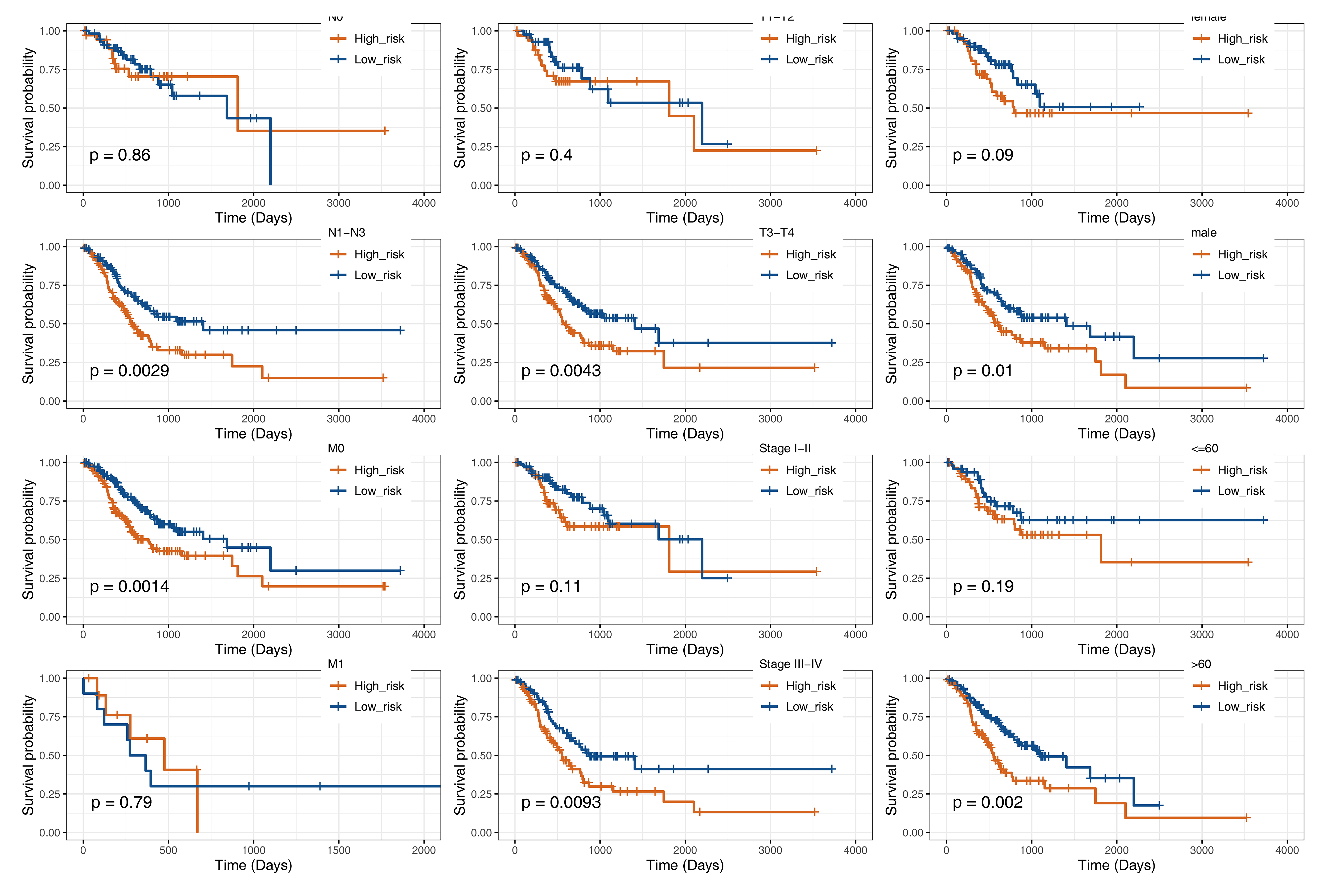

Supplement: Supplemental Information 3 [file peerj-12-16951-s003.png]

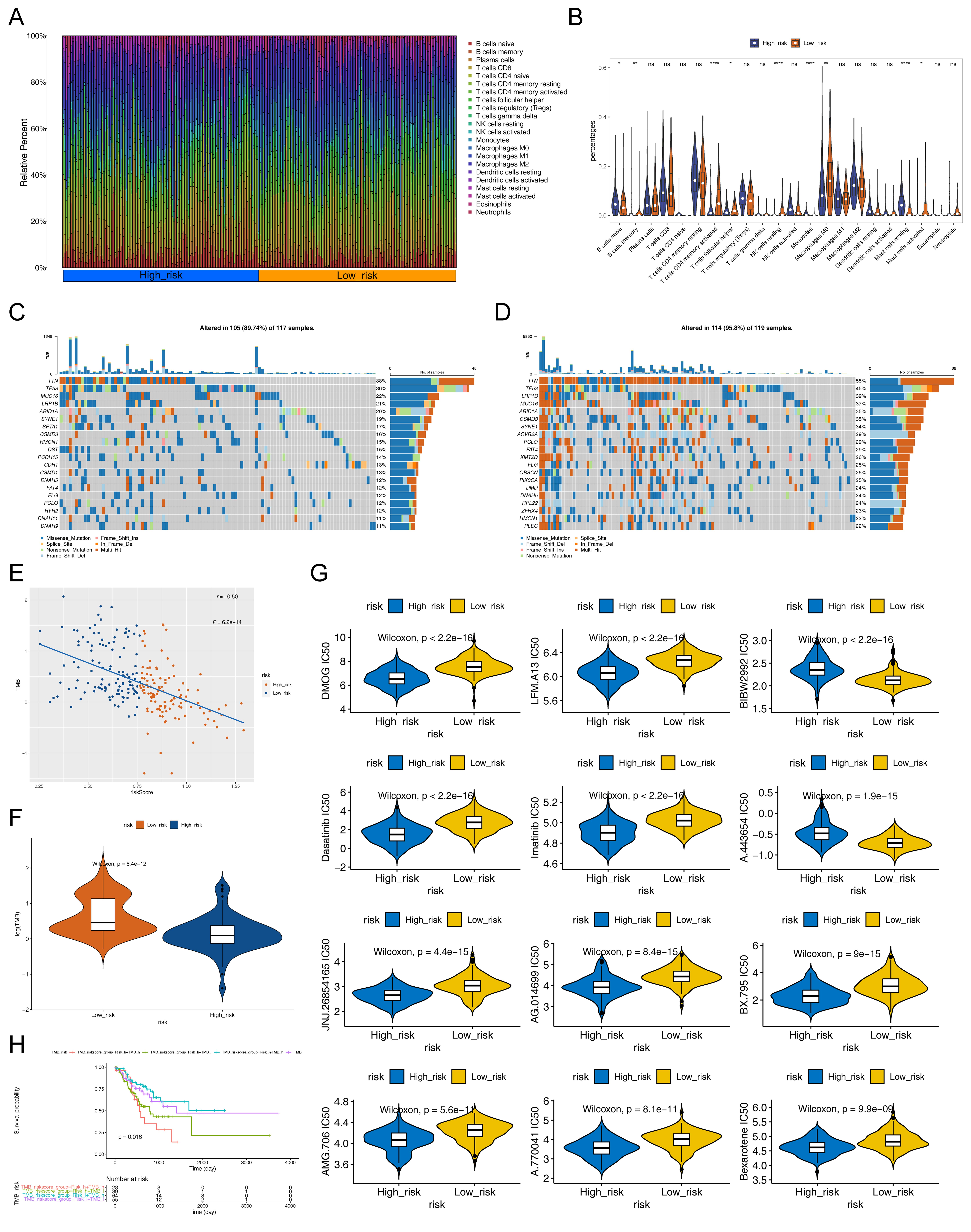

Supplement: Supplemental Information 4 [file peerj-12-16951-s004.png]

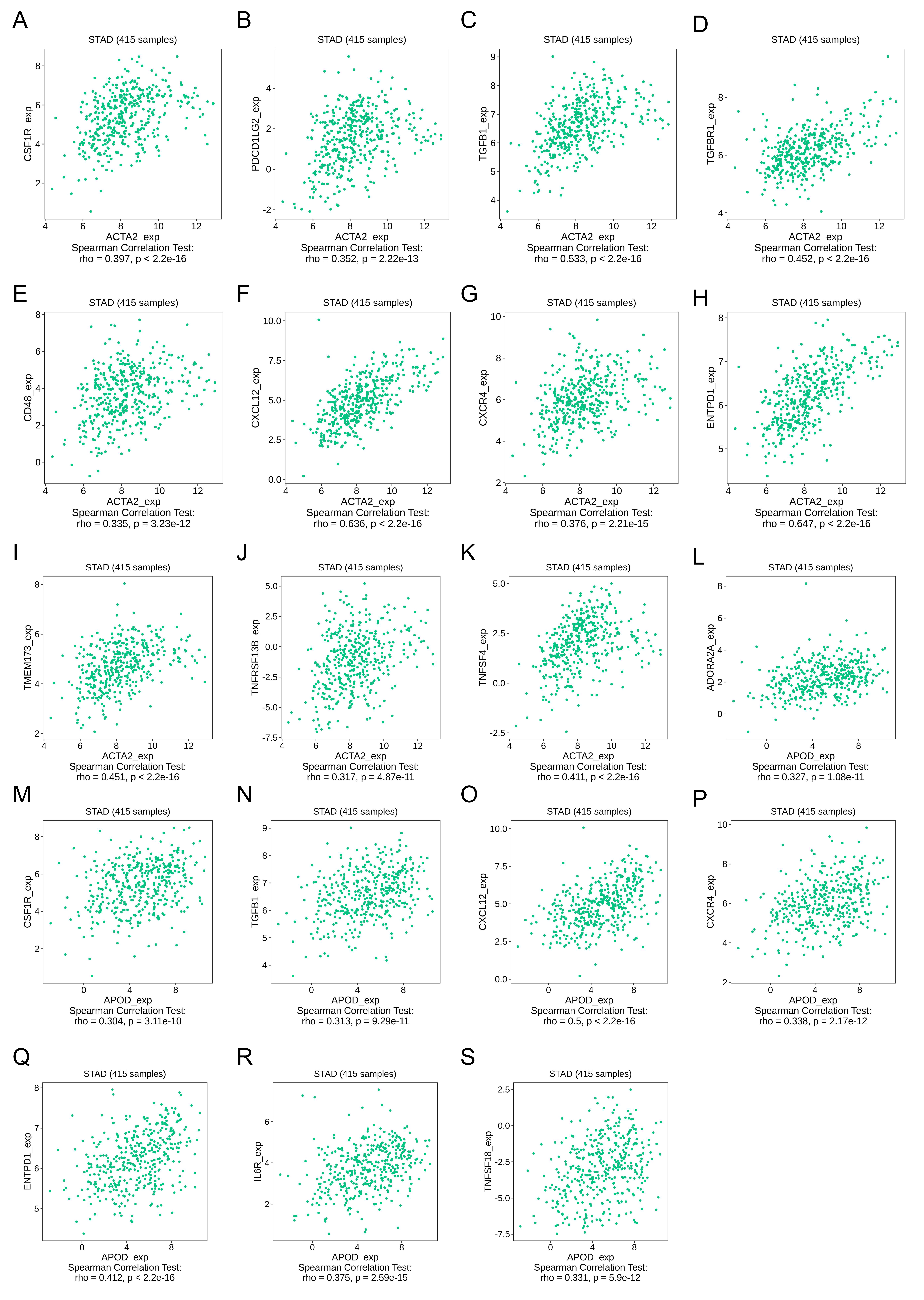

Supplement: Supplemental Information 5 [file peerj-12-16951-s005.png]

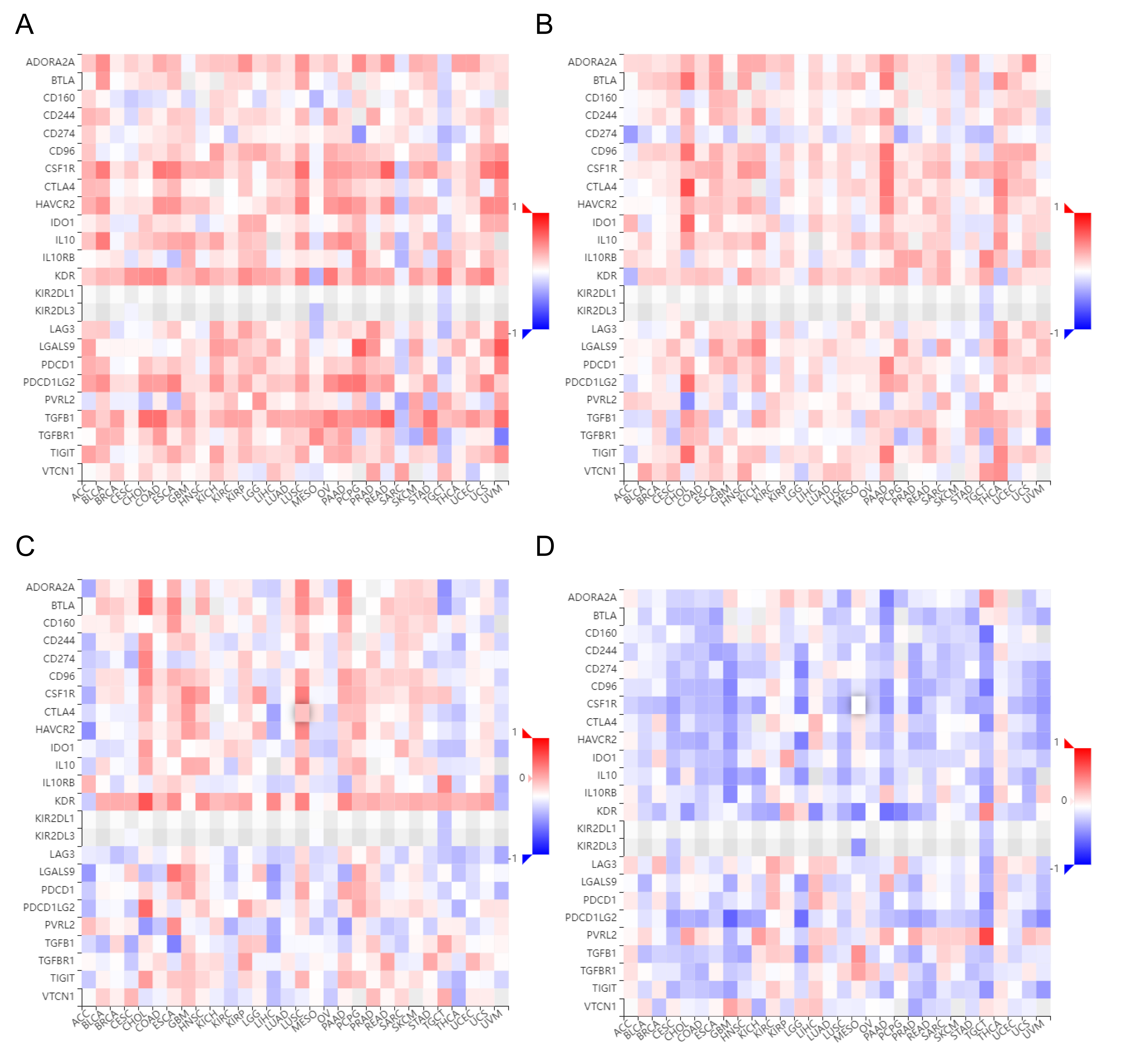

Supplement: Supplemental Information 6 — (A) Correlation between ACTA2 and immunoinhibitors. (B) Correlation between APOD and immunoinhibitors. (C) Correlation between APOD and immunoinhibitors. (D) Correlation between SAPCD2 and immunoinhibitors. [file peerj-12-16951-s006.png]

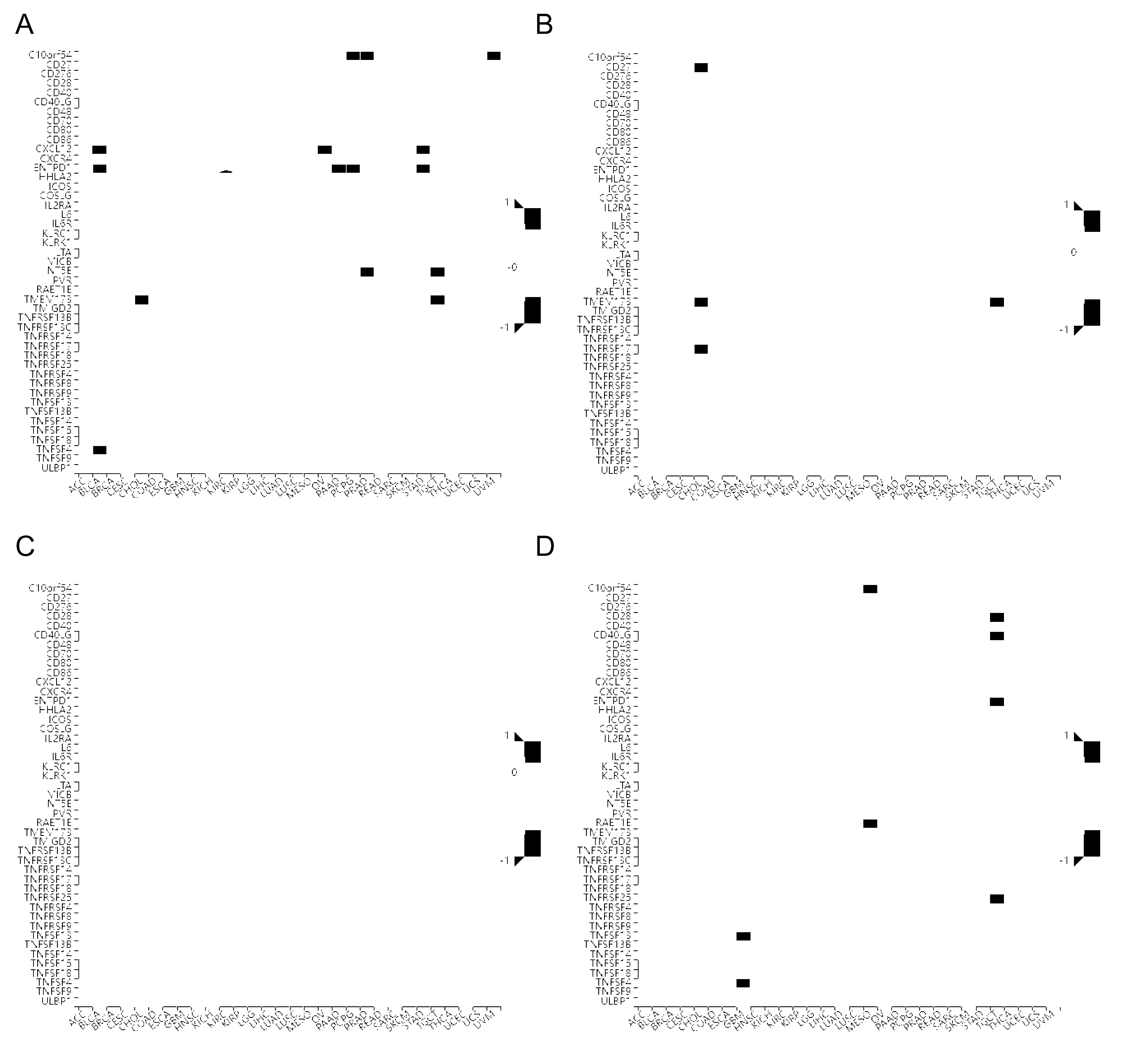

Supplement: Supplemental Information 7 — (A) Correlation between ACTA2 and immunostimulators. (B) Correlation between APOD and immunostimulators. (C) Correlation between APOD and immunostimulators. (D) Correlation between SAPCD2 and immunostimulators. [file peerj-12-16951-s007.png]

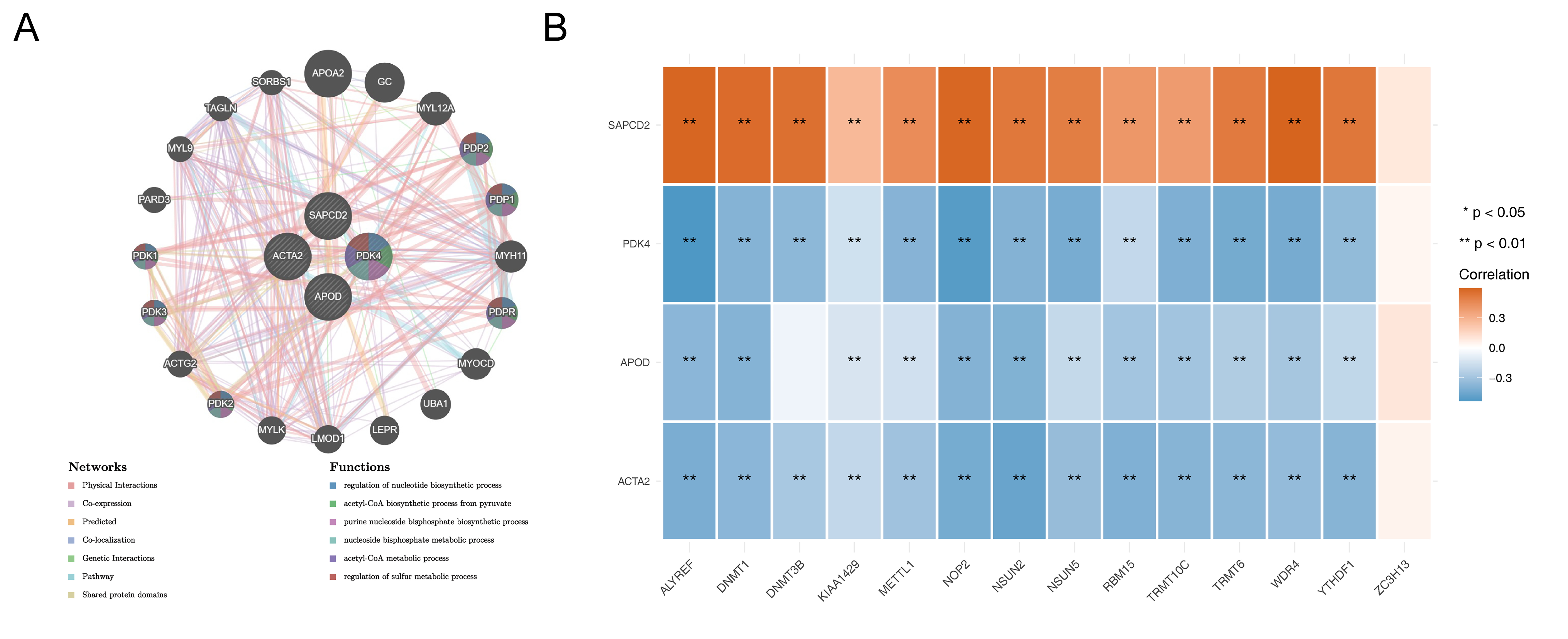

Supplement: Supplemental Information 9 — (A) The PPI network of prognostic genes. (B) The relevance of prognostic genes and DERMGs. **p < 0.01. [file peerj-12-16951-s009.png]

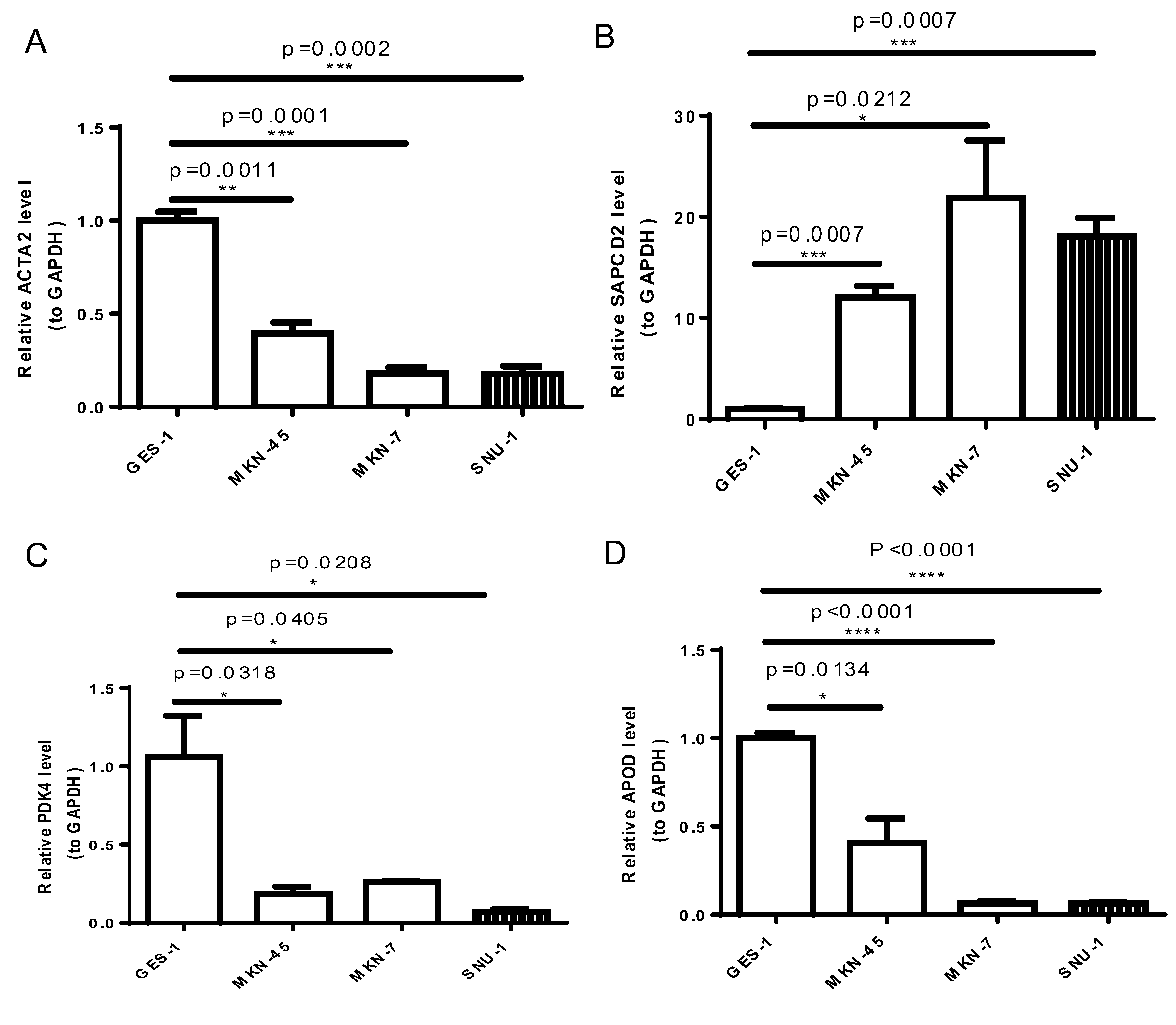

Supplement: Supplemental Information 10 — (A) ACTA2 (B) SAPCD2 (C) PDK4 (D) APOD *p < 0.05, **p < 0.01, ***p < 0.001, ****p < 0.0001. [file peerj-12-16951-s010.png]

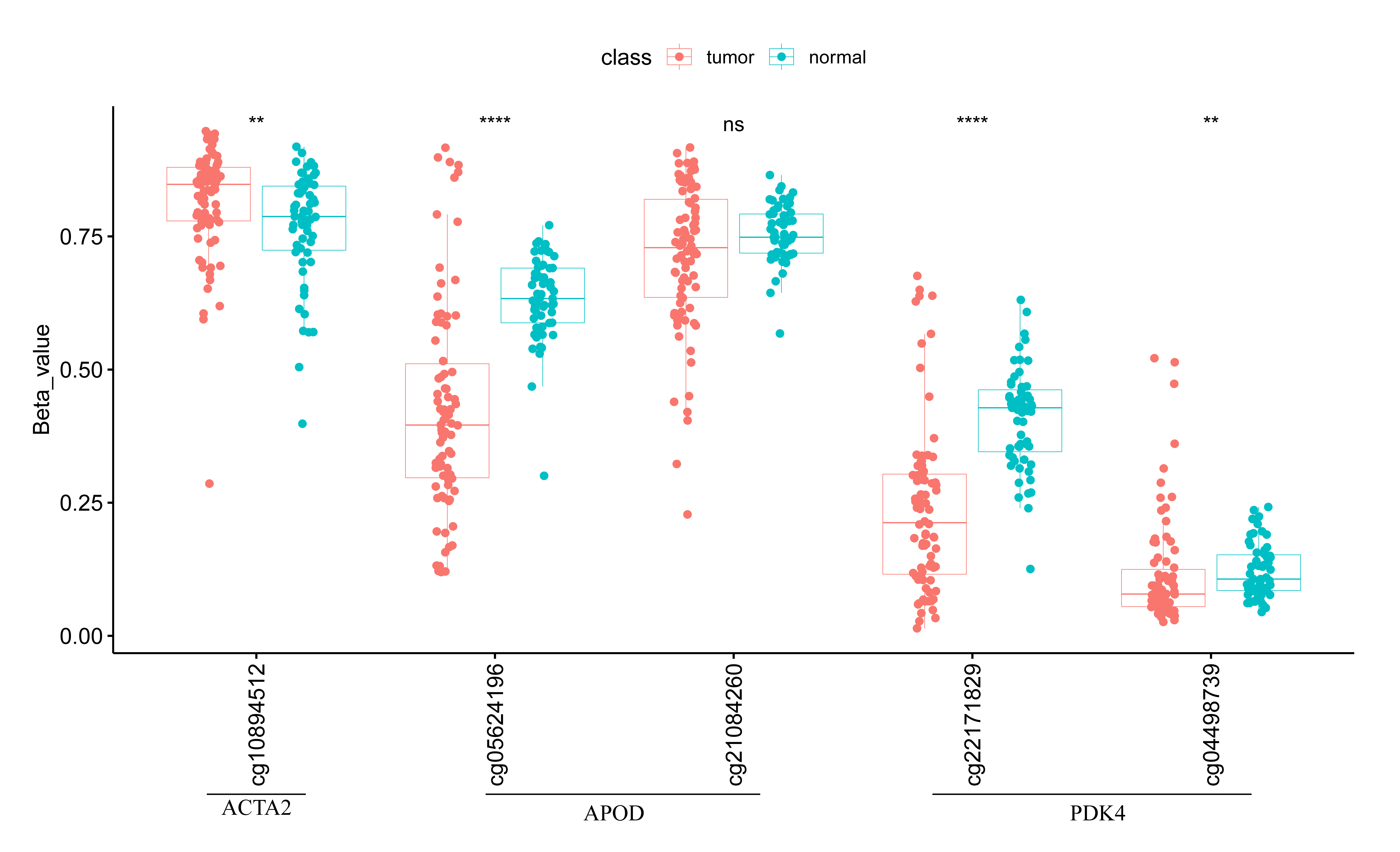

Supplement: Supplemental Information 11 [file peerj-12-16951-s011.png]
